# Advances in screening and detection of gastric cancer

**DOI:** 10.1002/jso.26844

**Published:** 2022-04-28

**Authors:** Jonathan Y. Xia, A. Aziz Aadam

**Affiliations:** ^1^ Division of Gastroenterology and Hepatology Northwestern University Feinberg School of Medicine Chicago Illinois USA

**Keywords:** detection, gastric cancer, screening

## Abstract

With an estimated one million new cases and 769 000 deaths in 2020, gastric cancer is the fifth most frequent cancer and fourth leading cause of cancer death globally. Incidence rates are highest in Asia and Eastern Europe. This manuscript will review the current modalities of diagnosis, staging, and screening of gastric cancer. We will also highlight development of novel diagnostics and advancements in endoscopic detection of early gastric cancer.

## 1 INTRODUCTION

Gastric cancer is the fifth most commonly diagnosed cancer worldwide and fourth most common cause of cancer related mortality.^1^ Rates are twofold higher in men than in women. Incidence rates are highest in Eastern Asia. In the United States, there are an estimated 26 000 new cases annually and 11 000 gastric cancer deaths each year.^2^ The overall 5‐year survival rate of gastric cancer is poor at 32.4%. This is likely due to the fact that up to 62% of gastric cancer cases in the United States are detected at advanced stages, which is associated with worse overall survival compared with localized disease.^2^

To improve the survival rate of gastric cancer, several nations with high prevalence of disease have established population‐based gastric cancer screening programs. Such screening methods resulted in improvement in early gastric cancer detection and ultimately increased overall survival.^3^, ^4^ Currently, there are no screening guidelines for gastric cancer in the United States. However, recent studies have found that individuals with lower socioeconomic status and racial/ethnic minorities are at increased risk for gastric cancer compared with non‐Hispanic white populations.^5^ Thus, there remains a potential role for targeted screening in high‐risk populations in the United States.

This manuscript will review the current modalities of diagnosis and staging of gastric cancer in the United States and worldwide. We will also examine the screening methods for gastric cancer that are currently implemented in East Asian countries. Finally, we will provide novel insights on the utility of targeted screening of high‐risk populations in the United States.

## 2 DIAGNOSIS OF GASTRIC CANCER

Gastric adenocarcinoma is classified into two anatomical subtypes, cardia (upper stomach adjoining the esophagus) and noncardia (mid and distal stomach). They differ in terms of risk factors, carcinogenesis, and epidemiological patterns.^1^ Cardia tumors have similar risk factors as esophageal adenocarcinoma that includes gastroesophageal reflux disease (GERD) and obesity while non‐cardia tumors are mainly preceded by atrophic gastritis and intestinal metaplasia.^6^, ^7^ Previous epidemiology studies have demonstrated that non‐cardia tumors continue to be diagnosed twice as often as cardia tumors and represent more than 80% of all gastric adenocarcinomas.^8^, ^9^, ^10^

The initial diagnosis of gastric cancer often is delayed because the majority of patients are asymptomatic during the early phases of disease. Weight loss and persistent abdominal pain are the most common symptoms at diagnosis.^11^ Occult gastrointestinal bleeding, with or without iron deficiency anemia is also a common symptom, while overt bleeding in the form of melena or hematemesis is seen in less than 20% of cases.^11^

Patients may also present with signs or symptoms of distant metastatic disease. Lymphatic spread may present with the classical findings of a left supraclavicular lymphadenopathy, Virchow's node, which is the most common physical examination finding of metastatic disease.^12^ Other signs of lymphatic spread include periumbilical lymphadenopathy, Sister Mary Joseph's Node, or left axillary lymphadenopathy, Irish Node.^12^ Findings of an enlarged ovary, Krukenberg tumor, or mass in the cul‐de‐sac on rectal examination, Blumer's shelf, are classical signs of peritoneal tumor spread.^13^, ^14^ More rarely, patients with gastric cancer may present with complications from direct extension of the mass through the gastric wall in the form of gastrocolic fistula or mechanical colonic obstruction.^14^ Furthermore, gastric cancer can also present with para‐neoplastic manifestations that include sudden appearance of diffuse sebohrrheic keratosis also known as sign of Leser‐Trelat, acanthosis nigricans, palmo‐plantar keratoderma, or hypercoagulable states.^14^, ^15^ Thus, in later stages of disease, gastric cancer can lead to the development of a wide variety of local and systemic signs and symptoms.

Patients presenting with high‐risk symptoms or multiple risk factors for gastric cancer usually require further work‐up. Serum tumor markers including CEA, CA‐125, CA 19‐9 may be elevated in patients with gastric cancer but they are of limited diagnostic utility due to their low sensitivity and specificity.^16^ An increase in serum pepsinogen II has been used for screening purposes to identify patients with increased risk for gastric cancer but it also lacks the sensitivity and specificity to provide any diagnostic value.^16^ Previously, double‐contrast barium swallow was the noninvasive study of choice in initial diagnostic work up.^17^ Barium studies can identify both malignant gastric ulcers and infiltrating lesions. However, false‐negative barium studies can occur as much as 50% of cases.^17^, ^18^ This is a particular problem in early gastric cancer where the sensitivity of barium studies maybe as low as 14%.^17^, ^18^ Endoscopy is used to analyze suspicious lesions identified on barium examination such as presence of stenosis, deformity, rigidity, indentation, filling defects in the wall, flattening and changes of gastric folds, barium pooling, or presence of polypoid lesion. Due to the current widespread availability of upper endoscopy and computed tomography (CT) scans, barium studies have become less popular as a diagnostic modality for gastric cancer.

Currently, esophagogastroduodenoscopy (EGD) is the diagnostic imaging procedure of choice in tissue diagnosis and tumor localization of gastric carcinoma. EGD is a highly sensitive and specific diagnostic test, especially when it is combined with endoscopic biopsy for tissue diagnosis.^19^ Gastric cancer typically appears endoscopically as a friable, ulcerated mass. In patients with endoscopic findings of gastric ulcer, the presence of nodular folds or thickened irregular margins are also suggestive of presence of malignancy.^19^ Since up to 5% of malignant ulcer grossly appear benign, any suspicious‐appearing gastric lesions or ulcerations found on upper endoscopy should be biopsied. A single biopsy has approximately 70% sensitivity for diagnosing an existing gastric cancer and sensitivity increases to 98% after seven biopsies along the ulcer margin and base.^19^ In patients with higher risk for gastric cancer, it is important to take numerous biopsies from suspicious‐appearing lesions and smaller, benign‐appearing gastric ulcers as diagnosis of early gastric cancer offers the greatest opportunity for cure and long‐term survival.

Once a tissue diagnosis of gastric cancer is obtained, the patient should undergo a complete staging evaluation to determine prognosis and guide therapy. The current more widely used staging system developed by the American Joint Committee on Cancer is the TNM staging criteria based on tumor, node, metastasis classifications.^20^ CT scans of the chest, abdomen, and pelvis are indicated in all patients with gastric cancer to evaluate for metastatic disease (M stage).^20^ For patients with gastric cancer who have no radiographic evidence of metastatic disease, endoscopic ultrasound (EUS) is recommended for assessment of depth of invasion of primary gastric cancers.^21^ Recent meta‐analysis that EUS has an overall high accuracy in assessing depth of invasion (T stage) with a sensitivity of 86%.^22^ Accurate evaluation of the depth of invasion is important in guidance of therapy toward resection alone or necessity of neoadjuvant chemotherapy. In addition, EUS with fine‐needle aspiration (FNA) can be used in conjunction with CT for evaluation of suspicious lymph nodes to increase accuracy of nodal staging.^23^

18‐fluorodexyglucose positron emission tomography (FDG‐PET) scan is also used for evaluation of distant metastases in patients with confirmed local disease. FDG‐PET has been shown to be more sensitive than CT for the detection of lymph node involvement and distant metastases.^24^ Diagnostic Laparoscopy can also be used for the detection of occult peritoneal dissemination in patients with more than a T1a lesion on EUS but no radiographic or histologic confirmation of stage IV disease. Although it is more invasive than other staging modalities, laparoscopy has the advantage of directly visualizing liver surface and the peritoneum and can be used for in depth examination of local lymph nodes. Previous studies have shown that 20‐30% of patients with disease beyond T1 stage on EUS will have peritoneal metastases despite a negative staging CT.^25^ Thus, the two most important factors influencing survival in patients with resectable gastric cancer are the depth of cancer invasion through the gastric wall and the number of lymph nodes involved.

## 3 GASTRIC CANCER SCREENING

The 5‐year survival of gastric cancer approaches 95%–99% when it is diagnosed at an early and resectable stage compared with less than 30% when diagnosed in advanced stages.^2^, ^26^, ^27^, ^28^ In East Asian countries such as Japan and Korea with high prevalence of gastric cancer, implementation of national screening guidelines for gastric cancer has proven be both cost‐effective and able to reduce gastric cancer related mortality.^29^ In Japan, radiographic screening via upper gastrointestinal series with barium meal was initiated locally in the 1960s and expanded nationwide in 1983.^4^ Radiographic screening was initially the only recommended method for gastric cancer screening due to limited evidence of mortality reduction of endoscopic screening. However, two Japanese case‐controlled studies that were published in 2013 demonstrated efficacy of mortality reduction in gastric cancer by endoscopic screening compared with no screening. One study conducted by Hamashima et al in Tottori and Niigata Prefectures of Japan comparing 410 patients diagnosed with gastric cancer and 2292 matched controls showed a 30% reduction in gastric cancer mortality by endoscopic screening compared with no screening within 36 months before the date of diagnosis.^30^ Another study conducted by Matsumoto et al evaluating 13 patients who died of gastric cancer between 2000 and 2008 and 130 controls showed 79% reduction of mortality from gastric cancer in patients who have undergone endoscopic screening within the previous 5 years before the date of diagnosis.^31^ These findings led to the use of upper endoscopy as a method of gastric cancer screening in 2014. The updated guidelines recommended that endoscopic screening be conducted in individuals 50 or older every 2 years.^4^ *Helicobacter pylori* antibody and serum pepsinogen levels have also been used at the local level in Japan as a combined method of screening but it is not recommended as a primary method of screening due to its high false‐positive rate.^4^, ^32^ In 2018, data from the Japanese Cancer Information Service showed that gastric cancer mortality in male and female decreased to 44.8 and 24.1 per 100 000 from 55.6 to 33.4 per 100 000 previously in 1958.^33^ Thus, in Japan, both radiographic and endoscopic screening is recommended to the general population for gastric cancer and it has been shown to be effective at decreasing gastric cancer related mortality.

Similar to Japan, South Korea implemented national gastric cancer screening in 1999 as part of its National Cancer Screening Program. It provided gastric cancer screening every 2 years for individuals 40 years or older and offered both radiographic and endoscopic screening modalities.^29^ Since its implementation, gastric cancer screening rates in South Korea increased from 7.4% in 2002% to 45.4% in 2011.^34^ In a nested case‐control study using the National Cancer Screening Program data for gastric cancer since 2002 consisting of 127 288 subjects with newly diagnosed gastric cancer, Jun et al showed that the screening program led to an overall 21% reduction in gastric cancer mortality.^29^ Furthermore, this study discovered a 47% reduction in gastric cancer mortality for individuals that were screened by upper endoscopy that was not observed for those that were screened with upper GI series.^29^ In addition, this study also showed an overall cumulative decrease in mortality risk from gastric cancer with increased episodes of endoscopic screening in individuals.^29^ Due to these findings, the use of radiographic screening has been removed from Korean guidelines for gastric cancer screening.

Although gastric cancer has relative low prevalence in the United States, there are high‐risk groups who could benefit from targeted screening programs. Regional population‐based studies in the United States have identified significant differences in the incidence of gastric cancer based on race/ethnicity. Dong et al.^5^ conducted a retrospective cohort study from 2008 to 2014 in Southern California demonstrated that Asians, Hispanics, and non‐Hispanic black populations were found to have up to 50% increased risk for gastric cancer compared with the non‐Hispanic white population. The same study also identified low socioeconomic status as an independent risk factor for gastric cancer.^5^ Consistent with these findings, Lui et al conducted a retrospective study examining over 55 000 gastric cancer cases from the National Cancer Institute's Surveillance, Epidemiology, and End Results (SEER) cancer registry from 1992 to 2009. They found overall gastric cancer incidence rate among Asians, Blacks, and Hispanics is more than double the rate among non‐Hispanic whites in the United States.^35^ Furthermore, a study by Saumoy et al. in 2018 demonstrated that endoscopic screening for gastric cancer in high‐risk racial and ethnic groups in the United States starting at age 50 years with surveillance every 3 years is cost‐effective.^36^ Two prior studies have also modeled the economic impact of gastric cancer screening in the United States and did not find it to be cost‐effective. However, neither study stratified according to race or ethnicity.^37^, ^38^ Due to these findings, the American Society of Gastrointestinal Endoscopy have recommended the consideration of gastric cancer screening with upper endoscopy among new US immigrants older than 40 years from high‐risk endemic regions including Japan, Korea, China, Russia, and South America. However, there were no recommendations regarding other high‐risk races and ethnicities in the United States, specifically Hispanics and non‐Hispanic blacks.^39^ Hopefully, the identification of racial and ethnic differences of gastric cancer incidence in the United States will stimulate efforts to address these differences through implementation of targeted screening in high‐risk populations.

## 4 EARLY GASTRIC CANCER

National screening programs in Japan and Korea has facilitated early detection of gastric malignancies, resulting in the establishment of the concept of early gastric cancer (EGC). At its initial conception in 1971 by the Japanese Society of Gastroenterology and Endoscopy, EGC was a gastric neoplasm that could be successfully treated with surgery.^40^ Currently, EGC is defined as a gastric adenocarcinoma that invades no deeper than the submucosa. With the introduction of screening programs, the proportion of EGC rose from 15 to about 57% in Japan. In, a case series of patients diagnosed with EGC and followed without surgery, 63% of tumors progressed to advanced carcinomas over a span of 6–88 months.^40^ The identification of EGC has profoundly impacted clinical management of gastric cancer as studies have shown that patients that undergo resection of EGC have a 5‐year survival rate of >90%.^41^, ^42^ In addition, the need for better endoscopic identification of EGC has driven the development of novel imaging technologies for early neoplasia detection such as narrow band imaging and autofluorescence imaging. Furthermore, the need for better management approaches of EGC has led to the development of advanced endoscopic resection techniques such as endoscopic submucosal dissection (ESD).^43^

In Asia, ESD has become the standard of care for the management of EGC meeting the absolute and expanded indications by the Japanese Gastric Cancer Association. Initially, the Japanese Gastric Cancer Association proposed that non‐ulcerated EGCs confined to the mucosa (T1a) and <2 cm as absolute indication for ESD due to their low risk for lymph node metastasis.^44^ ESD provided the advantage of en bloc resection of EGC, which previously could not be achieved with endoscopic mucosal resection (EMR) if lesions were greater than 2 cm. Furthermore, ESD has proven to be superior compared with EMR in the management of EGC in terms of having both reduced local recurrence of lesions and improved 5‐year disease‐free survival.^43^ Studies comparing radical gastrectomy and ESD for the management of EGC has shown that ESD is associated with almost 50% reduction in postoperative morbidity and decreased hospital stay while having similar 3‐year survival.^43^ In light of the excellent outcomes achieved with ESD, the indications for ESD were subsequently expanded to include (1) non‐ulcerated EGCs of any size, (2) ulcerated differentiated EGCs <3 cm, or (3) differentiated EGCs <3 cm with superficial submucosal invasion. A large multicenter study in 2017 by Tanabe et al comparing outcomes of ESD in patients that met either absolute or expanded criteria showed a recurrence rate of 1.26% in the 4202 patients who met the expanded criteria compared with 0.22% in patients who met the absolute criteria.^26^ Metastatic recurrence was found to be 0.7% in patients that met expanded criteria when compared with 0.2% in patients meeting absolute criteria.^26^ Similarly, Tate et al. in 2019 studied outcomes of 135 ESD cases meeting either absolute or expanded criteria from a single center with a Western population and showed only 2 cases of recurrence and high rates of curative resections. Thus, ESD is associated with an extremely low rate of disease recurrence in patients with EGC meeting both absolute and expanded criteria in both Eastern and Western patient populations.

## 5 NOVEL DIAGNOSTICS AND DETECTION METHODS OF EARLY GASTRIC CANCER

Recent advances in next generation sequencing have facilitated improved understanding of the molecular pathogenesis of gastric cancer leading to identification of novel biomarkers for early gastric cancer diagnosis. When tumor cells grow, they can release nucleic acids such as DNA and RNA into the blood, thus, making circulating tumor DNA (ctDNA), microRNAs (miRNAs), long noncoding RNAs (lncRNA), and circular RNA (circRNA) all promising noninvasive methods in early‐diagnosis of gastric cancer. Compared to previously mentioned protein‐based tumor markers (CEA, CA 19‐9, etc.), these novel biomarkers offer both improved sensitivity and specificity. Several pilot studies have shown that ctDNA can differentiate patients with gastric cancer and healthy individuals with significantly improved sensitivity and specificity compared with conventional biomarkers.^45^, ^46^, ^47^ Interestingly, patients with early stage (surgically resectable) disease had lower serum burden of ctDNA along with lower number of genetic alterations in the ctDNA itself.^47^ MiRNAs are small noncoding RNAs that have been found to be deregulated in preneoplastic conditions such as atrophic gastritis and intestinal metaplasia and in early gastric dysplasia.^48^ Specific miRNAs such as miRNA‐21 and miR‐376c have been found to be upregulated in serum of patients with early stages of gastric cancer and has positive predictive value of up to 90%.^48^ Similarly, lncRNA and circRNA are new classes of noncoding RNAs identified through RNA sequencing that have shown to be associated with tumor growth and metastasis. Studies have shown that lncRNA and circDNA levels in serum can not only detect presence of early gastric cancer but also be used to help monitor depth of gastric cancer invasion and presence of lymphatic metastasis.^48^ Larger prospective studies are needed to validate these novel promising circulating molecules as reliable biomarkers for early gastric cancer. Developing highly sensitive and specific biomarkers for gastric cancer would be integral in enhancing early disease diagnosis and survival in countries with low incidence of disease, such as Western countries, where massive population screening strategies are not cost‐effective.

The disparity in regional gastric cancer prevalence results in significant variations in endoscopic experience and technical expertise in the detection of early gastric cancer. Past studies have shown that gastric cancers and precursor lesions are frequently missed in upper endoscopy by underexperienced endoscopists and detection rate can be improved after training and use of narrow band imaging (NBI).^49^, ^50^ Recent implementation of artificial intelligence (AI) and deep learning in endoscopy have demonstrated that AI can be successfully used in assisting in detection of colorectal polyps, predicting Barrett's neoplasia, and improving endoscopy quality.^51^, ^52^, ^53^ Similarly, a team in China has developed an AI system, ENDOANGEL‐LD, through training a deep learning algorithm with retrospective and real time endoscopic images from over 10 000 patients across 6 different hospitals.^54^ This system has been demonstrated to exhibit a sensitivity and specificity of over 90% in detection of early gastric when trialed prospectively in over 2000 patients.^54^ Another Korean based group developed a similar model using AI to detect not only gastric mucosal lesions but also estimate depth of lesion invasion. This model, called AI‐Scope, was shown to have superior performance in detection of gastric lesions compared with novice and intermediate endoscopists and similar performance with experts. Interestingly, the AI‐Scope model estimated the invasion depth of EGC better than endoscopic ultrasound. Thus, these advancements in deep learning algorithms provide the framework of future models where AI can be used as a companion technology to aid endoscopists in detection of early gastric cancer.

## 6 CONCLUSION AND FUTURE DIRECTIONS

Overall, gastric cancer incidence has steadily declined in the United States over the past 50 years. This is mainly related to the identification and eradication of *H. pylori* infections in the population. However, recent epidemiology studies have shown that annual incidence has increased from 0.27 to 0.45 per 100 000 for the non‐Hispanic white population ages 25–39 years.^55^ In addition, studies have shown that gastric cancer risk is 50% increased in racial/ethnic minority populations when compared with their non‐Hispanic white counterparts in the United States. The identification of the concept of EGC has led to novel diagnostic and therapeutic advances that are being used to improve outcomes of gastric cancer and other diseases throughout the gastrointestinal tract. Furthermore, advancements in development of novel biomarkers and AI holds the potential of earlier and improved detection of gastric cancer. These findings combined with the excellent outcomes of the management of EGC makes a case for gastric cancer screening in the new high‐risk populations in the United States.

## SYNOPSIS

Advancements in endoscopy, imaging, and development of novel biomarkers along with artificial intelligence hold the potential for earlier and improved detection of gastric cancer.

## Data Availability

The data availability statement is not available.
